# Characterizing Marathon-Induced Metabolic Changes Using ^1^H-NMR Metabolomics

**DOI:** 10.3390/metabo11100656

**Published:** 2021-09-27

**Authors:** Rachelle Bester, Zinandré Stander, Shayne Mason, Karen M. Keane, Glyn Howatson, Tom Clifford, Emma J. Stevenson, Du Toit Loots

**Affiliations:** 1 Human Metabolomics, Faculty of Natural and Agricultural Sciences, North-West University, Private Bag X6001, Box 269, Potchefstroom 2531, South Africa; rachelle05.rb@gmail.com (R.B.); stander.zinandre@mayo.edu (Z.S.); nmr.nwu@gmail.com (S.M.); 2 Department of Sport Exercise and Nutrition, School of Science and Computing, Galway Mayo Institute of Technology, H91 T8NW Galway, Ireland; karen.keane@gmit.ie; 3 Faculty of Health and Life Sciences, Department of Sport, Exercise and Rehabilitation, Northumbria University, Newcastle upon Tyne NE1 8ST, UK; glyn.howatson@northumbria.ac.uk; 4 Water Research Group, School of Environmental Sciences and Development, North-West University, Potchefstroom 2531, South Africa; 5 School of Sport, Exercise and Health Sciences, Loughborough University, Loughborough LE11 3TU, UK; t.clifford@lboro.ac.uk; 6 Human Nutrition Research Centre, Faculty of Medical Sciences, Newcastle University, Newcastle upon Tyne NE2 4HH, UK; Emma.Stevenson@newcastle.ac.uk

**Keywords:** endurance races, marathon, metabolites, untargeted metabolomics, ^1^H-NMR spectrometry, serum metabolome

## Abstract

Although physical activity is a health-promoting, popular global pastime, regular engagement in strenuous exercises, such as long-distance endurance running races, has been associated with a variety of detrimental physiological and immunological health effects. The resulting altered physiological state has previously been associated with fluctuations in various key metabolite concentrations; however, limited literature exists pertaining to the global/holistic metabolic changes that are induced by such. This investigation subsequently aims at elucidating the metabolic changes induced by a marathon by employing an untargeted proton nuclear magnetic resonance (^1^H-NMR) spectrometry metabolomics approach. A principal component analysis (PCA) plot revealed a natural differentiation between pre- and post-marathon metabolic profiles of the 30-athlete cohort, where 17 metabolite fluctuations were deemed to be statistically significant. These included reduced concentrations of various amino acids (AA) along with elevated concentrations of ketone bodies, glycolysis, tricarboxylic acid (TCA) cycle, and AA catabolism intermediates. Moreover, elevated concentrations of creatinine and creatine in the post-marathon group supports previous findings of marathon-induced muscle damage. Collectively, the results of this investigation characterize the strenuous metabolic load induced by a marathon and the consequential regulation of main energy-producing pathways to accommodate this, and a better description of the cause of the physiological changes seen after the completion of a marathon.

## 1. Introduction

The year 2021 marks the 125th anniversary of the first marathon run during the 1896 Summer Olympics in Greece. The popularity of this event sparked the conception of long-distance (≥5 km) endurance running races, generally categorized as half-marathons (21.1 km), marathons (42.2 km) and ultra-marathons (≥42.2 km) [1]. Not only has participation in marathons become increasingly common, but it has also become affiliated with the many health benefits that are associated with aerobic exercise [2]. The most notable of which is the lower prevalence of cardiovascular disease [3], elevated cognitive health [4], and increases in skeletal muscle mitochondrial volume as well as subsequent increases in muscle oxidative capacity [5]. There is, however, a large disparity between the energy expenditure and bodily demands associated with marathon running in comparison to most other aerobic exercises. As such, regular participation in these endurance running events have been found to induce numerous potentially deleterious immunological and physiological health effects. Some of these immunological effects include acute pro- and anti-inflammatory responses [6], damage to bronchial epithelial cells [7], a perturbed mucosal immune system, and higher susceptibility to symptoms of upper respiratory tract infections [8]. On a physiological level, short-term occurrences of muscle damage [9] and medial tibial stress syndrome [10] are common for the average marathon participant, while more acute effects such as an increased risk for myocardial fibrosis [11], deleterious cardiac structural changes [12], as well as acute liver and renal damage [13] have been reported for extreme/elite veteran marathon athletes.

Although these immunological and physiological effects have been well characterized, there is limited literature on the impact of these races on metabolite fluctuations (metabolome) of marathon runners using untargeted metabolomics. Metabolomics aims to comprehensively detect, identify, and quantify fluctuations in metabolite (<1500 Da organic and inorganic chemical compounds) concentrations in a biological system in response to a perturbation (disease, environmental factors, drug-intake, lifestyle, dietary, etc.), as a means of providing information regarding the altered physical state [14,15,16]. 

Previous investigations [17,18,19,20] that have employed targeted and/or semi-targeted metabolomics approaches have provided credible information on the metabolic effects of strenuous exercises. In short, energy production takes place in a hierarchical manner during physical activity [21], where the contribution of each metabolic pathway is determined by factors such as the overall intensity, duration, and frequency of exercise [22]. The various metabolic processes at play include: (1) substrate-level phosphorylation via the phosphocreatine system [23], providing sufficient ATP for only a few seconds of running activity [18]; (2) anaerobic glycolysis and homolactic fermentation of pyruvic acid, producing sufficient ATP for an additional few minutes of running activity [22]; and (3) aerobic catabolism of dietary substrates by means of oxidative phosphorylation, which is of high energetic value to endurance athletes, since it is able to supply sufficient ATP to support several hours of exercise, provided sufficient nutrient store availability [22]. Carbohydrates are widely recognized as the primary aerobic ATP source utilized during endurance running events [18,24,25], although the capacity of aerobic glycolysis can be limited during continuous running activity [26]. Insufficient free glucose and glycogen stores reportedly lead to a gluconeogenic influx, reduced insulin secretion, and an elevated glucagon/insulin ratio, subsequently activating alternative energy-producing pathways such as lipolysis and protein catabolism [25].

According to Hawley and Leckey [27], the aerobic carbohydrate utilization rate is reduced, while an upregulated fatty acid oxidation is observed in skeletal muscles during endurance exercise [28]. This is supported by various previous metabolomics studies [18,20,24,29] that observed elevated concentrations of fatty acids, glycerol, acyl-carnitines, and ketone bodies, concurrent with upregulated lipolysis and ketogenesis activity. Moreover, saturation of beta-oxidation (elevated 3-hydroxy acids) and subsequent upregulation of omega-oxidation (elevated dicarboxylic acids), which is normally considered to be a minor pathway capable of compensating for incomplete beta-oxidation, has been reported [24,25] following a marathon. In addition to the utilization of lipids as an alternative fuel substrate, a general reduction in amino acids (AA) and elevation in their associated catabolism intermediates has also been observed in previous metabolomics investigations [19,20,24], further indicating the utilization of proteins/AA as yet another alternative fuel substrate. 

Although previous studies provide credible information pertaining to endurance exercise-induced metabolic changes, most are based on studies done using targeted and/or semi-targeted approaches (biased) that were performed in controlled environments (cycling, treadmill, rowing activities) [30]. As such, the current study is aimed at investigating the effects of a marathon (42.2 km) on the serum metabolome of 30 recreational marathon runners by using an untargeted proton nuclear magnetic resonance (^1^H-NMR) metabolomics approach. Considering this, we aim to not only confirm the previously proposed marathon-induced metabolic changes, but to possibly identify additionally affected metabolic pathways, allowing for a more holistic view of the global metabolome change induced by a marathon.

## 2. Results

**Figure 1:**
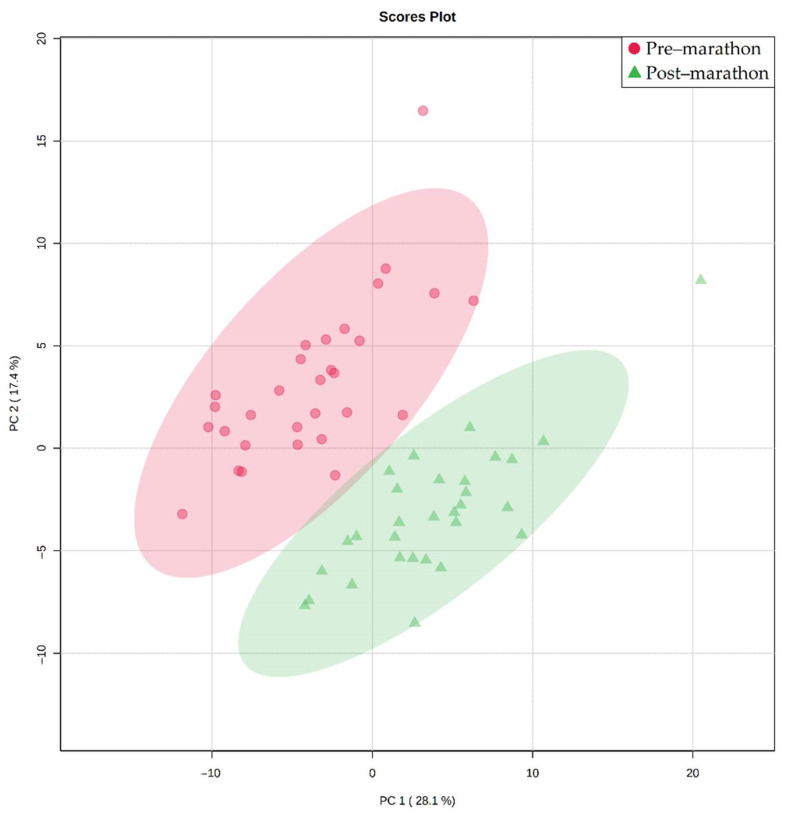
Principal component analysis (PCA) plot illustrating the natural differentiation of the pre-marathon (red circles) and post-marathon (green triangles) serum metabolic profiles of the 30 marathon participants.

The principal component analysis (PCA) plot (Figure 1) shows clear separation of the pre-marathon and post-marathon metabolome data. Upon employing the first round of the multi-statistical approaches, 67 of the original 132 ^1^H-NMR spectral bins were deemed significant, while the second round identified 17 statistically significant metabolites associated with these bins. These metabolites are listed in Table 1, and fluctuations are discussed in detail thereafter (associated PCA loading plot is illustrated in Figure S1).

## 3. Discussion

**Figure 2:**
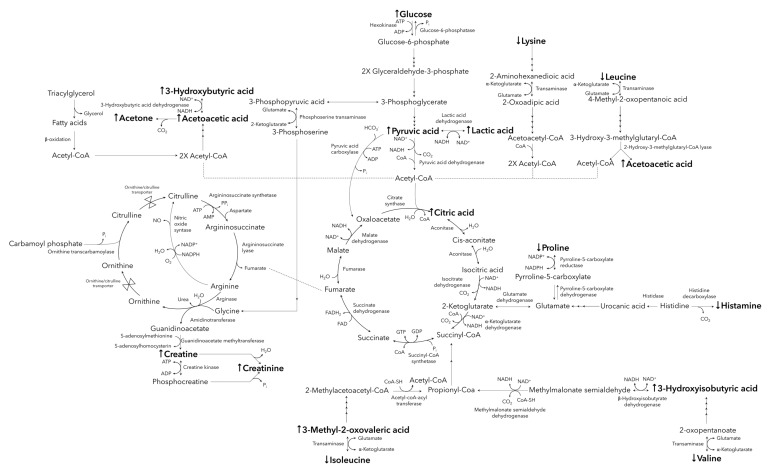
A schematic description of the marathon-induced metabolic changes, showing increased (↑) and decreased (↓) concentrations of significantly altered metabolites in the post-marathon samples (**in bold text**) are indicated relative to the pre-marathon values. ATP adenosine triphosphate, ADP adenosine diphosphate, AMP adenosine monophosphate, NAD^+^ nicotinamide adenine dinucleotide, NADH reduced nicotinamide adenine dinucleotide, NADP^+^ nicotinamide adenine dinucleotide phosphate, NADPH reduced nicotinamide adenine dinucleotide phosphate, P_i_ inorganic phosphate, CoA coenzyme A, FAD flavin adenine dinucleotide, FADH_2_ reduced flavin adenine dinucleotide, GTP guanosine triphosphate, GDP guanosine diphosphate.

The majority of the metabolites listed in Table 1 are indicative of changes to the main energy-producing pathways, including the phosphagen system, anaerobic and aerobic glycolysis, the tricarboxylic acid (TCA cycle), ketogenesis, and amino acid oxidation (illustrated in Figure 2).

Anaerobic glycolysis typically involves the conversion of accumulating pyruvic acid to lactic acid, via lactic acid dehydrogenase, accepting NADH as a coenzyme, and producing NAD^+^ [31]. This is concurrent with the elevated post-marathon lactic acid and pyruvic acid observed in the current investigation (Figure 2) and is further supported by previous studies [18,25]. Although this mechanism provides a more rapid method of energy production than aerobic glycolysis and aids in the maintenance of the NAD^+^/NADH ratios [31], its performance is restricted due to the resulting lactic acidosis [32], hence coercing the transition to aerobic glycolysis and the catabolism of alternative fuel substrates [22].

It is well known that carbohydrates are preferentially oxidized by the body during endurance-type exercises [23], reportedly leading to glucose and glycogen store “depletion” within approximately 90 min after the start of endurance running (at >75% of maximum oxygen uptake) [26]. However, elevated serum glucose was observed immediately post-marathon in this investigation (Figure 2). This is supported by the studies conducted by Stander et al. [25] and Lewis et al. [18] who reported elevated post-marathon serum glucose, as well as an elevation in the gluconeogenesis-associated metabolites. A plausible explanation for this includes the initial depletion of free glucose as well as intramuscular and liver glycogen stores, resulting in downregulated insulin secretion, upregulated gluconeogenesis, and an elevated glucagon/insulin ratio [25]. This phenomenon is thought to be regulated by a variety of factors, including altered hormone secretion (glucocorticoids) in response to the stress signals caused by the hypoxic state and the strenuous energy demands induced during the endurance race [33]. Cortisol is one of the major glucocorticoids associated with the latter, and results in the translocation of glucose transporters to the cell membrane, subsequently inhibiting glucose uptake during fasting and/or exercising and eventuating elevated blood glucose levels [34].

Endurance-induced adaptations (generally only reported for highly trained aerobic athletes) of the skeletal muscles includes a slower utilization of carbohydrates and an upregulated lipid metabolism [27]. Although the current study cohort includes both amateur and well-trained marathon participants, no significant differences were observed when comparing the respective metabolic profiles based on previous endurance running experience. Lipids are also catabolized (especially 60–90 min into such endurance events) via β-oxidation, contributing to the production of acetyl-CoA [28], and initially resulting in an upregulated channeling of the latter into the TCA-cycle. This acetyl-CoA influx may account for the elevated serum concentration of citric acid observed during the current (Figure 2) and previous studies [18,25]. Additionally, the high energy demands and associated imbalanced redox state induced by participation in such activities [35] may cause an upregulation in citric acid synthase and pyruvic acid dehydrogenase activity, as previously observed by McKenzie et al. [36], in an attempt to produce the much-needed NADH/FADH_2_ and, ultimately, ATP via the electron transport chain. However, the continuous influx of acetyl-CoA from the various energy-producing pathways, accompanied by the aforementioned imbalanced redox state, may exceed the mitochondrial oxidative capacity, eventuating in the activation of ketogenesis [31,37]. The latter is demonstrated in this investigation by the elevated concentrations of 3-hydroxybutyric acid, acetone, and acetoacetic acid observed in the post-marathon serum samples (Figure 2).

In accordance with previous literature [38], AA catabolism was activated as an alternative means of producing energy during the marathon (Figure 2). This is supported by a reduction in concentrations of AAs (leucine, isoleucine, valine, lysine, and proline) and the elevation of the various observed serum AA catabolism intermediates (3-methyl-2-oxovaleric acid and 3-hydroxyisobutyric acid). Additionally, the reduced concentrations of serum histamine (decarboxylated form of histidine) observed in the post-marathon samples (Figure 2) may be ascribed to the preferred catabolism of histidine for ATP synthesis via the TCA cycle, rather than to be decarboxylated to histamine [39]. Lastly, considering the role of histamine during acute inflammatory responses, reduced post-marathon histamine may additionally be ascribed to an immune suppression experienced during the “open window effect” directly after the marathon [39,40].

Although protein catabolism normally only contributes to supplying a small amount of the total energy requirements during a marathon, branched-chain amino acids (BCAA) are preferentially oxidized [25], a situation triggered by, amongst others, a reduced ATP:ADP ratio, acidosis, and the “depletion” of muscle glycogen stores [36]. Furthermore, the reduced post-marathon serum concentrations of leucine are known to inhibit glutamine transport into the cells, subsequently inhibiting mTORC1 and resulting in autophagy [25] as the body’s last resort to find the necessary energy-producing substrates to comply with the massive energy demands required to complete such an event [41].

Lastly, the elevated serum levels of creatine and creatinine, are most likely indicative of muscle damage [42], or perhaps also to a lesser extent, a declining kidney function [43], or myocardial cell injury [44], all of which have been previously proposed to potentially occur during strenuous endurance exercise.

In conclusion, the current study was aimed at investigating marathon-induced (42.2 km) metabolite shift using an untargeted ^1^H-NMR metabolomics approach. The aforementioned metabolic changes to aerobic and anaerobic glycolysis, ketogenesis, AA catabolism (in particular, BCAAs) and the TCA cycle, indicated the extent to which the body needs to adapt in order to comply with the energy demands required for the completion of a marathon. Increases in all three endogenous ketone bodies and decreases in all three BCAAs reflect a high reliance on their associated metabolic pathways for energy production, suggesting a possible target for the development of athletic performance-enhancing strategies. The decreased post-marathon histamine concentration has not been reported before and may suggest an alternative source of energy production during a marathon run. Furthermore, the presence of creatinine and creatine in post-marathon samples primarily supports the occurrence of exercise-induced muscle damage. The next step would be to use these findings towards more effective recovery and athletic performance-enhancing strategies, which target those specific energy-producing metabolic pathways shown to be drastically altered in this study.

One of the most apparent confounding factors of this investigation, as in the case of all human-based studies, is the unavoidable presence of inter-individual variability. In an attempt to compensate for the latter, the study design pertinently included paired measures of the participants, thus allowing each participant to serve as their own control. Nonetheless, inter-individual variation, as well as the uncontrolled environmental setting of the study, allows for a higher level of robustness of the findings, considering that the true nature of the marathon-perturbation is represented. Future investigations may consider using larger sample cohorts to further support the results obtained here, and to repeat the current study in a variety of alternative geological locations with differences in climate, humidity, altitude, and atmospheric pressure to elucidate the underlying causative relationship between environmental factors and metabolic adaptations. Results generated from this investigation provide a basis for further, more targeted and/or semi-targeted metabolomics approaches that may aim to correlate the metabolite fluctuations, perhaps with varying running distances, speed, and athlete experience.

## 4. Materials and Methods

### 4.1. Participants

Volunteers provided written informed consent prior to participation. Participant eligibility was assessed by completing a health screening questionnaire, in which individuals with food allergies, cardiovascular complications, musculoskeletal disorders/injuries, and those receiving anti-inflammatory treatment were excluded from the study. Female athletes were required to complete a menstrual cycle questionnaire, and all participants were instructed to record their dietary intake from 24 h preceding pre-marathon sampling, up to 48 h post-marathon. Based upon these exclusion criteria, 30 marathon runners were included in this study. A summary of participant characteristics is provided in Table 2. Ethical approval was obtained from the North-West University Health Research Ethics Committee (ethics number: NWU-00163-21-A1).

### 4.2. Druridge Bay Marathon

The marathon took place in 2016 and entailed 4 laps around the Druridge Bay country park, located on the Northumberland coast (Morpeth, UK). The route was mainly flat and included a combination of paved and grassy terrain, as well as approximately 6.4 km (1.6 km per lap) of soft sand on the coastline. The race started at 09:00, at which time the ambient temperature was 3.8 °C, wind speed 9 km h^−1^, humidity 82%, and barometric pressure 1013 hPa. At the end of the race (approximately 13:30) the ambient temperature and wind speed had increased to 8.5 °C and 14 km h^−1^, respectively, while the humidity decreased to 62%. Throughout the race, the weather remained mostly cloudy, with occasional sunshine.

### 4.3. Sample Collection and Storage

The current investigation forms part of a larger multidisciplinary collaboration study wherein physiological, immunological [45], and metabolic [25,46] analyses on subgroups of the current sample cohort have been performed and may be referred to for further information. Blood samples from 30 marathon runners were collected via antecubital fossa venesection of the basilica vein, before (pre-marathon) and immediately after (post-marathon) completion of a marathon run. In the week preceding the marathon, runners were required to be in a hydrated yet fasted state for 10 mL pre-marathon blood sample in the laboratory. Post-marathon samples were taken in the field at the finish line of the marathon within 1 h post-race before being placed on ice and transported to the Faculty of Health and Life Sciences, Department of Sport, Exercise, and Rehabilitation at Northumbria University in Newcastle, United Kingdom. Blood samples were then allowed to coagulate for 30 min before being centrifuged at 3000× *g* for 10 min. The supernatant/serum was extracted and immediately frozen at −80 °C, before being transported (on dry ice) to the North-West University, Human Metabolomics: Laboratory of Infectious and Acquired Diseases, South Africa. Samples were kept at −80 °C until metabolomics analyses were performed.

### 4.4. ^1^H-NMR Serum Buffer Solution

A 1.5 M buffer solution was prepared by dissolving 20.4 g potassium dihydrogen phosphate (KH_2_PO_4_) in 80 mL of deuterated water (D_2_O). Hereafter, 100 mg of trimethylsilylpropionic acid (TSP; internal standard) and 13 mg of sodium azide (NaN_3_) were dissolved in 6–10 mL of D_2_O. These two solutions were then combined and vortexed before pH adjustment to 7.4 via potassium hydroxide (KOH) pellets addition. Finally, the solution was transferred to a volumetric flask and the volume was adjusted to 100 mL with D_2_O.

### 4.5. Sample Preparation and Randomization

Prior to sample preparation, all samples were randomized and equally divided into 3 batches. Serum samples contain macromolecules, such as lipids and proteins, that may lead to spectral interference and poor spectral baselines, subsequently resulting in inaccurate identification and quantification of metabolites, if not removed. As such, all batched samples, including the pooled quality control (QC) samples (containing 50 µL of each test sample) were filtered using pre-rinsed (thrice with HPLC-grade H_2_O via centrifugation at 6000× *g* for 10 min) centrifugal filter units (10,000 Da filter pore size). A miniaturized ^1^H-NMR method, adapted from Mason et al. [47], was employed due to limited sample volumes. Briefly, 100 μL of each serum sample was pipetted onto the pre-rinsed centrifugal filters and centrifuged at 6000× *g* for 20 min. Hereafter, 6 μL of buffer solution and 54 μL of sample filtrate (10:90% buffer:sample ratio) were dispensed into 2 mm ^1^H-NMR tubes (outside diameter 2.0 mm, inside diameter 1.6 mm, length 100 mm) by using an eVol^®^ (Supelco, St. Louis, MO, USA) NMR automated digital syringe system (100 μL syringe and 180 mm long bevel-tipped needle) with a pre-loaded/programmed pipetting sequence. This mixture was homogenized by first aspirating, then dispensing the 60 μL solution back into the 2 mm ^1^H-NMR tubes. The syringe was washed three times between each sample transfer with distilled water. Employing the MATCH system (Bruker, Rheinstetten, Germany), samples were loaded onto a SampleXpress autosampler (Bruker, Rheinstetten, Germany) based on previous randomization, with QC samples set to be analyzed at the beginning, middle, and end of each batch for quality assurance purposes (Figures S2 and S3).

### 4.6. ^1^H-NMR Analysis

^1^H-NMR spectroscopy is a highly specific analytical platform with the capability to elucidate complex structural and conformational data from a wide variety of chemical classes [48]. The prepared serum samples, along with appropriate QC samples, were analyzed on a Bruker Avance III HD 500 MHz NMR spectrometer, equipped with a 5 mm triple-resonance inverse (TXI) probe head, which was kept at a constant temperature of 310 K (37 °C). In order to produce reproducible data, the following experimental parameter adjustments were made by utilizing Topspin (version 3.5, Bruker, Rheinstetten, Germany) prior to each sample analyzed: (1) shimming to the TSP signal was applied to correct for magnetic field inhomogeneity caused by variations of the applied magnetic field, as a result of imperfections in the main magnet or due to the presence of interfering compounds in the sample itself [49]; (2) the signal was automatically locked to a pre-defined D_2_O reference signal present in each sample in order to compensate for magnetic field drift [50]; and (3) the probe head was tuned to 500.133 MHz and the pulse was calibrated to ensure a resonant frequency at 90°. Each scan (*n* = 128) was subjected to an excitation pulse of 90° for 8 μs followed by a 4 s relaxation delay. Spectral width for the ^1^H-NMR spectra was 6000 Hz (12.0 ppm).

### 4.7. Data Processing and Clean-Up

Data pre-processing steps were automatically completed by Bruker Topspin (version 3.5) software and included: (1) Fourier transformation of the raw free induction decay signal to readable spectral peaks; (2) baseline phasing and correction; (3) TSP calibration to exactly 0.00 ppm; and (4) pre-saturation/suppression of H_2_O resonance at 4.72 ppm by single-frequency irradiation during the 4 s relaxation delay with 8 µs 90° excitation pulse, using NOESY-presat pulse sequence program. Moreover, spectral resolution was manually checked in order to ensure that shimming was done correctly by assessing that the width of the TSP peak, at half the height of the peak, was <1 Hz.

Further data processing steps were conducted using AMIX (version 3.9.14, Bruker, Rheinstetten, Germany), where the dataset was normalized relative to the internal standard (TSP), and the spectral data quantified across 132 bins (variable-sized binning). The advantage of binning used here was that no spectral regions of noise were included in the statistical analyses as noise can have a negative impact on principal component analysis [51]. Data clean-up steps included log-transformation using natural shift log transformation [52] (heteroscedasticity correction for non-gaussian variable distribution), as well as auto-scaling to align and correlate all variables [53], all of which were executed utilizing MetaboAnalyst (version 5.0, Xia research group, Saint Anne de Bellevue, QC, Canada) [54].

### 4.8. Bins/Metabolite Marker Selection and Statistical Analysis

Following data processing, the binned data was uploaded onto the MetaboAnalyst (version 5.0) software. Untargeted ^1^H-NMR metabolite selection proceeded in a biphasic manner. The first phase consisted of untargeted/unbiased statistical analysis to identify the bins significant pertaining to the aim of this investigation, while the more targeted, second phase identified the metabolites associated with the bins selected in phase one (multiple bins could be representative of one compound) that are significant to the aim of the investigation [52]. Although the multi-statistical approach employed included both univariate and multivariate methods, metabolites/bins were selected based on univariate methods only.

Univariate analyses included an independent effect size (Glass’s Δ effect size calculation as described by Ialongo [55]) and a paired t-test (corrected for multiple testing by the Benjamini-Hochburg procedure [56]), which was performed using Excel 2016 (Microsoft 365, version 2108) and MetaboAnalyst (version 5.0) [54], respectively. Additionally, multivariate analyses included a PCA, indicating whether a natural differentiation occurred between comparative groups.

In the case of the preliminary untargeted statistical bins selection (phase one), 132 bins were subjected to the basis of a large effect size (*d*-value ≥ 0.8) and an adjusted *p*-value cut-off lower than 0.05. After the first round, 67 bins were identified as statistically significant, and their respective peaks were identified using pure chemical compounds. ^1^H-NMR assignments are presented in Table 3. Following identification, only metabolites with a *d*-value ≥ 0.5 and *p*-value *≤* 0.05 were selected for interpretation during the second phase of statistical analyses. Finally, this allowed for the identification of 17 statistically significant metabolite markers listed in Table 1.

### 4.9. 2D-NMR Analysis and Identification

Homonuclear correlation spectroscopy (COSY) and homonuclear J-resolved spectroscopy (JRES) were used to produce two-dimensional NMR spectra for high confidence metabolite-identity confirmational purposes by increasing metabolite specificity through deconvolution techniques [57]. Two-dimensional COSY and JRES spectra were recorded with a spectral width of 8000 Hz in both dimensions, at 16 scans per increment, a recycle delay of 2 s, and a pulse of 8.5 µs (Figures S5–S9). Correlations between the acquired 2D-NMR spectra and ^1^H-NMR spectra, during which identical experimental conditions were followed, allows for level 1 confidence identification of non-novel metabolites [58].

### 4.10. Absolute Quantification

Following metabolite identification and confirmation through 2D COSY and/or JRES NMR analyses (Figures S5–S9), quantification was performed on corresponding peaks (Table 3), which had minimal overlaps and good signal to noise ratios (Figure S4). A feature unique to ^1^H-NMR analysis is the ability of the platform to produce spectra wherein the peak areas are directly proportional to the number of protons (nuclei) responsible for the peak. As a result, ^1^H-NMR-based quantification processes do not require the construction of a calibration curve based on pure compounds, and metabolites can be quantified provided that the signal area per proton is known [52]. This was achieved by the addition of a known concentration (0.5805 mM) of internal standard (TSP) to each sample. The signal area per proton was calculated by dividing the peak integral of TSP by the number of protons present in the molecule (H^+^ = 9). Identified metabolites were subjected to an identical procedure before equating each integral relative to TSP. By multiplying this value with the known concentration of TSP, the identified metabolites could be quantified in an absolute manner.

## Figures and Tables

**Table 1:** Statistically significant marathon-induced metabolite changes.

Metabolite (PubChem ID)	Pre-Marathon	Post-Marathon	Pre- vs. Post-Marathon	
	Average Concentration in µM (Standard Deviation)		*p*-Value (<0.05)	*d*-Value (≥0.5)
3-Hydroxybutyric acid (441) ^c^	56.7 (32.4)	424.8 (268.8)	1.0 $\times 10^{-12}$	3.7
3-Hydroxyisobutyric acid (87) ^a^ *	19.4 (5.9)	38.5 (9.4)	9.1 $\times 10^{-10}$	1.9
3-Methyl-2-oxovaleric acid (47) ^b^	35.7 (16.3)	70.5 (18.2)	7.7 $\times 10^{-8}$	1.4
Acetoacetic acid (96) ^b^	21.3 (6.2)	55.0 (26.2)	2.4 $\times 10^{-8}$	2.5
Acetone (180) ^b^	6.7 (2.1)	17.2 (11.2)	7.7 $\times 10^{-7}$	2.3
Citric acid (311) ^c^	137.5 (33.6)	221.9 (55.0)	2.9 $\times 10^{-10}$	2.0
Creatine (586) ^b^	67.9 (21.9)	100.2 (51.8)	9.7 $\times 10^{-5}$	1.1
Creatinine (588) ^b^	50.7 (9.5)	70.1 (18.5)	2.5 $\times 10^{-7}$	1.3
Glucose (5793)	1426.1 (382.1)	1927.3 (469.6)	1.4 $\times 10^{-5}$	1.1
Histamine (774) ^a^ *	93.9 (26.6)	68.9 (26.8)	2.5 $\times 10^{-3}$	1.5
Isoleucine (6306)	72.3 (20.4)	49.5 (10.9)	1.7 $\times 10^{-8}$	1.1
Lactic acid (612)	2472.0 (851.5)	4423.3 (1182.7)	2.0 $\times 10^{-8}$	1.9
Leucine (6106)	159.0 (36.8)	119.0 (22.0)	1.7 $\times 10^{-8}$	1.2
Lysine (5962)	161.4 (42.0)	127.6 (30.4)	1.4 $\times 10^{-5}$	0.9
Proline (145742) ^c^	284.1 (73.3)	219.2 (59.0)	5.8 $\times 10^{-7}$	1.0
Pyruvic acid (1060) ^b^	60.9 (28.1)	112.5 (38.5)	6.3 $\times 10^{-8}$	1.4
Valine (6287)	267.0 (53.3)	200.3 (35.1)	1.8 $\times 10^{-10}$	1.3

^a^ No JRES or COSY confirmation; ^b^ JRES 2D confirmation only; ^c^ COSY 2D confirmation only; * level 2 identification.

**Table 2:** Participant demographic information.

Participant Characteristics	Average ± Standard Deviation
Age (years)	41 ± 12
Gender (M/F)	18/12
Height (m)	1.7 ± 0.1
Mass change (kg)	−1.3 ± 1.0
Experience (years)	9.6 ± 8.4
Finishing time (hh:mm:ss)	04:16:13 ± 00:47:01

**Table 3:** ^1^H-NMR assignments of identified metabolites.

Peak	Metabolite	Chemical Shift (ppm)	Protons (n)	Multiplicity	Chemical Moiety
1	3-Hydroxybutyric acid ^c^	1.21	3	d	CH_3_
2	3-Hydroxyisobutyric acid ^a^ *	1.08	3	d	CH_3_
3	3-Methyl-2-oxovaleric acid ^b^	1.10	3	d	CH_3_
4	Acetoacetic acid ^b^	2.28	3	s	CH_3_
5	Acetone ^b^	2.24	6	s	CH_3_
6	Citric acid ^c^	2.60	2	d	CH_2_
7	Creatine ^b^	3.93	2	s	CH_2_
8	Creatinine ^b^	4.06	2	s	CH_2_
9	α-Glucose	5.24	1	d	CH
10	β-Glucose	4.66	1	d	CH
11	Histamine ^a *^	7.06	1	s	CH
12	Isoleucine	1.01	3	d	CH_3_
13	Lactic acid	1.33	3	d	CH_3_
14	Leucine	0.96	6	dd	(CH_3_)_2_
15	Lysine	3.02	2	t	CH_2_
16	Proline	2.01	2	m	CH_2_
17	Pyruvic acid ^b^	2.38	3	s	CH_3_
18	Valine	1.04	3	d	CH_3_

Peak numbers correspond to the labels used in Figure S4. ^a^ No JRES or COSY confirmation; ^b^ JRES 2D confirmation only; ^c^ COSY 2D confirmation only; * level 2 identification; s singlet; d doublet; dd double doublet; t triplet; m multiplet.

## Data Availability

The current study forms part of a multidisciplinary collaboration study consisting of numerous aims, each of which are being drafted into various manuscripts. As such, the datasets presented in this study are not publicly available but are available on request from the corresponding author. All authors declare that the presented results within this study are a clear and honest representation of the data without fabrication, falsification, or inappropriate data manipulation.

## Supplementary Materials

The following are available online at https://www.mdpi.com/article/10.3390/metabo11100656/s1, Figure S1: PCA loading plot of all identified metabolites significantly influenced by a marathon perturbation, Figure S2: PCA plot indicating the clustering of QC samples, demonstrating the absence of a batch effect, Figure S3: Inter-batch and intra-batch repeatability of all ^1^H-NMR bins in QC samples prior to statistical analyses. FDA suggested 20% CV cut-off related to, 79% (intra-batch repeatability) and 86% (inter-batch repeatability) of all bins, while selecting a 50% CV cut-off, 93% (intra-batch repeatability) and 96% (inter-batch repeatability) of all bins fell within range, Figure S4: 1D ^1^H-NMR spectrum of QC sample with important metabolites identified. 1 = 3-Hydroxybutyric acid (1.21d, J = 6.2 Hz), 2 = 3-Hydroxyisobutyric acid (1.08d, J = 7.0 Hz), 3 = 3-Methyl-2-oxovaleric acid (0.90t, J = 7.4 Hz; 1.10d, J = 7.0 Hz), 4 = Acetoacetic acid (2.28s), 5 = Acetone (2.24s), 6 = Citric acid (2.60AB, J = 15.3 Hz), Creatine (3.04s; 3.93s), Creatinine (3.05s; 4.06s), 9 = α-Glucose (5.24d, J = 3.7 Hz), 10 = β-Glucose (4.66d, 7.9 Hz), 11 = Histamine (7.06s, 7.79d), 12 = Isoleucine (0.94t, J = 7.4 Hz; 1.01d, J = 7.0 Hz), 13 = Lactic acid (1.33d, J = 7.0 Hz; 4.12q, J = 6.9 Hz), 14 = Leucine (0.96dd, J = 5.9 Hz), 15 = Lysine (1.73m; 1.91m; 3.02t, J = 7.6 Hz), 16 = Proline (2.01m), 17 = Pyruvic acid (2.38s), 18 = Valine (0.99d, J = 7.0 Hz; 1.04d, J = 7.0 Hz), Figure S5: 1D ^1^H-NMR and 2D ^1^H-^1^H JRES NMR confirmation of metabolites using pure compound library. Metabolites: 3-hydroxybutyric acid, 3-hydroxyisobutyric acid, 3-methyl-2-oxovaleric acid, isoleucine, valine, and leucine, Figure S6: 1D ^1^H-NMR and 2D ^1^H-^1^H JRES NMR confirmation of metabolites using pure compound library. Metabolites: glucose and lactic acid, Figure S7: 1D ^1^H-NMR and 2D ^1^H-^1^H JRES NMR confirmation of metabolites using pure compound library. Metabolites: creatinine and creatine, Figure S8: 1D ^1^H-NMR and 2D ^1^H-^1^H JRES NMR confirmation of metabolites using pure compound library. Metabolites: lysine, proline, pyruvic acid, acetone, and acetoacetic acid, Figure S9: 2D ^1^H-^1^H COSY NMR confirmation of metabolites using pure compound library. Metabolites: 3-hydroxybutyric acid, citric acid, glucose, isoleucine, leucine, valine, lysine, proline, and lactic acid.
