# Supplementary material for: Characterizing Marathon-Induced Metabolic Changes Using 1H-NMR Metabolomics
Source: Metabolites. 2021 Sep 27;11(10):656. doi: 10.3390/metabo11100656 (PMC8541139; doi:10.3390/metabo11100656)
Supplement: Supplementary file 1 [file metabolites-11-00656-s001.zip › metabolites-1360828-supplementary.pdf]

Supporting Information

Characterizing Marathon-Induced Metabolic Changes Using ^{1}H-NMR Metabolomics

Rachelle Bester[1], Zinandré Stander[1], Shayne Mason[1], Karen M. Keane[2], Glyn Howatson[3,4], Tom Clifford[5], Emma J. Stevenson[6] and Du Toit Loots[1,*]

[1] Human Metabolomics, Faculty of Natural and Agricultural Sciences, North-West University, Private Bag X6001, Box 269, Potchefstroom 2531, South Africa; rachelle05.rb@gmail.com (R.B.); stander.zinandre@mayo.edu (Z.S.); nmr.nwu@gmail.com (S.M.)

2 Department of Sport Exercise and Nutrition, School of Science and Computing, Galway Mayo Institute of Technology, H91 T8NW Galway, Ireland; karen.keane@gmit.ie

3 Faculty of Health and Life Sciences, Department of Sport, Exercise and Rehabilitation, Northumbria University, Newcastle upon Tyne NE1 8ST, UK; glyn.howatson@northumbria.ac.uk

4 Water Research Group, School of Environmental Sciences and Development, North-West University, Potchefstroom 2531, South Africa

5 School of Sport, Exercise and Health Sciences, Loughborough University, Leicestershire LE11 3TU, UK; t.clifford@lboro.ac.uk

6 Human Nutrition Research Centre, Faculty of Medical Sciences, Newcastle University, Newcastle upon Tyne NE2 4HH, UK; Emma.Stevenson@newcastle.ac.uk

* Correspondence: dutoit.loots@nwu.ac.za

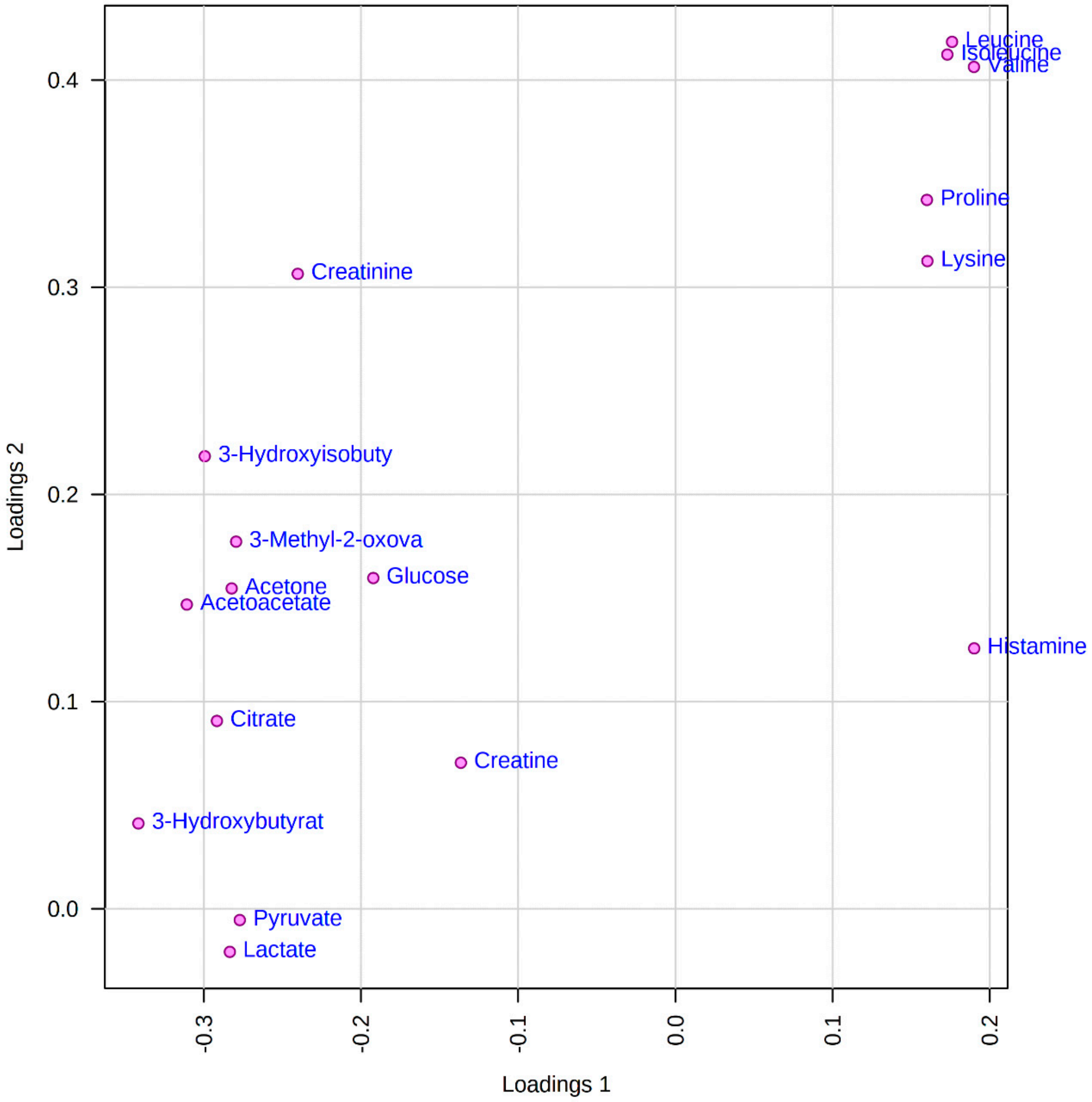

Figure S1. PCA loading plot of all identified metabolites significantly influenced by a marathon perturbation.

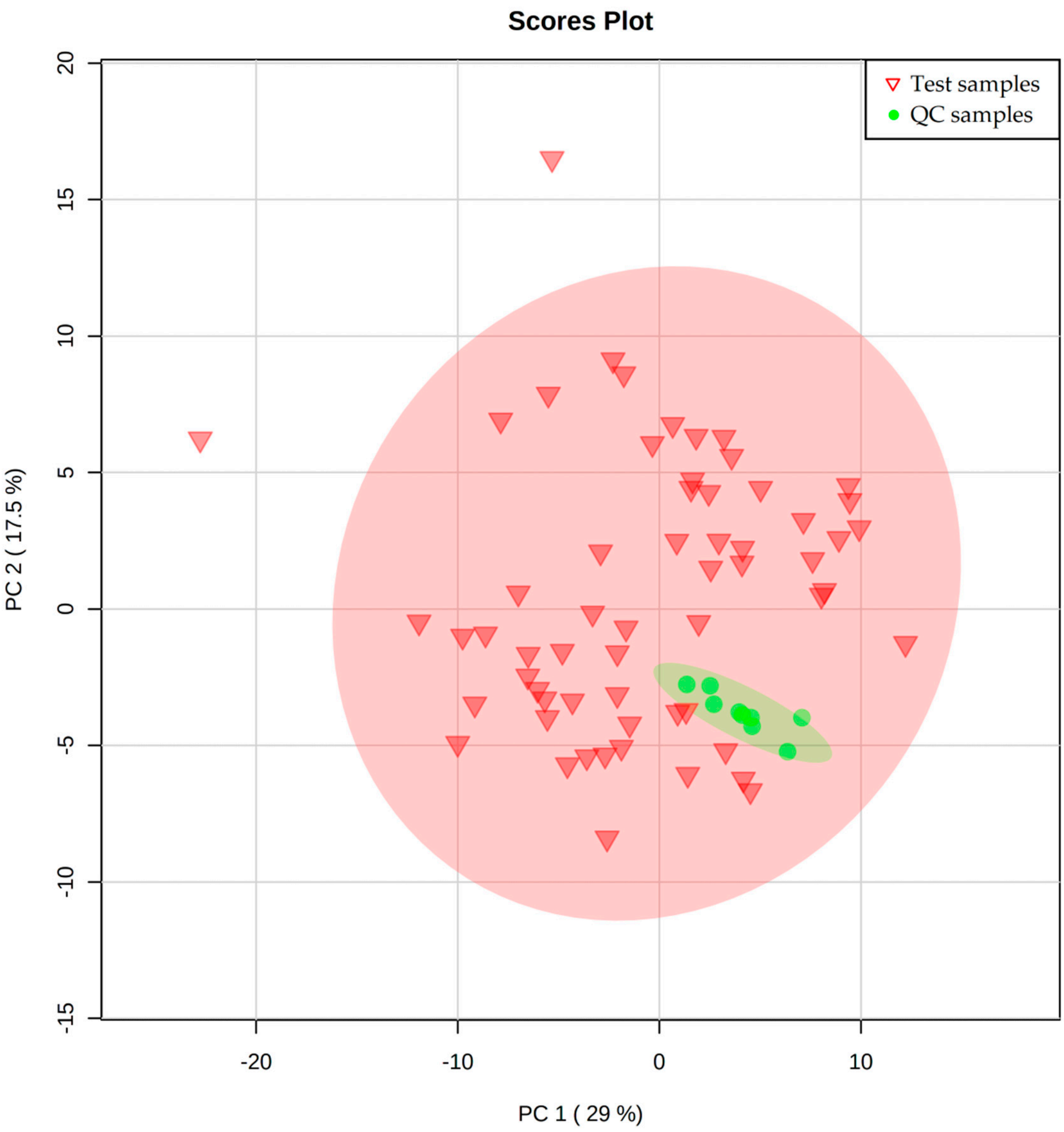

Figure S2. PCA plot indicating the clustering of QC samples, demonstrating the absence of a batch effect.

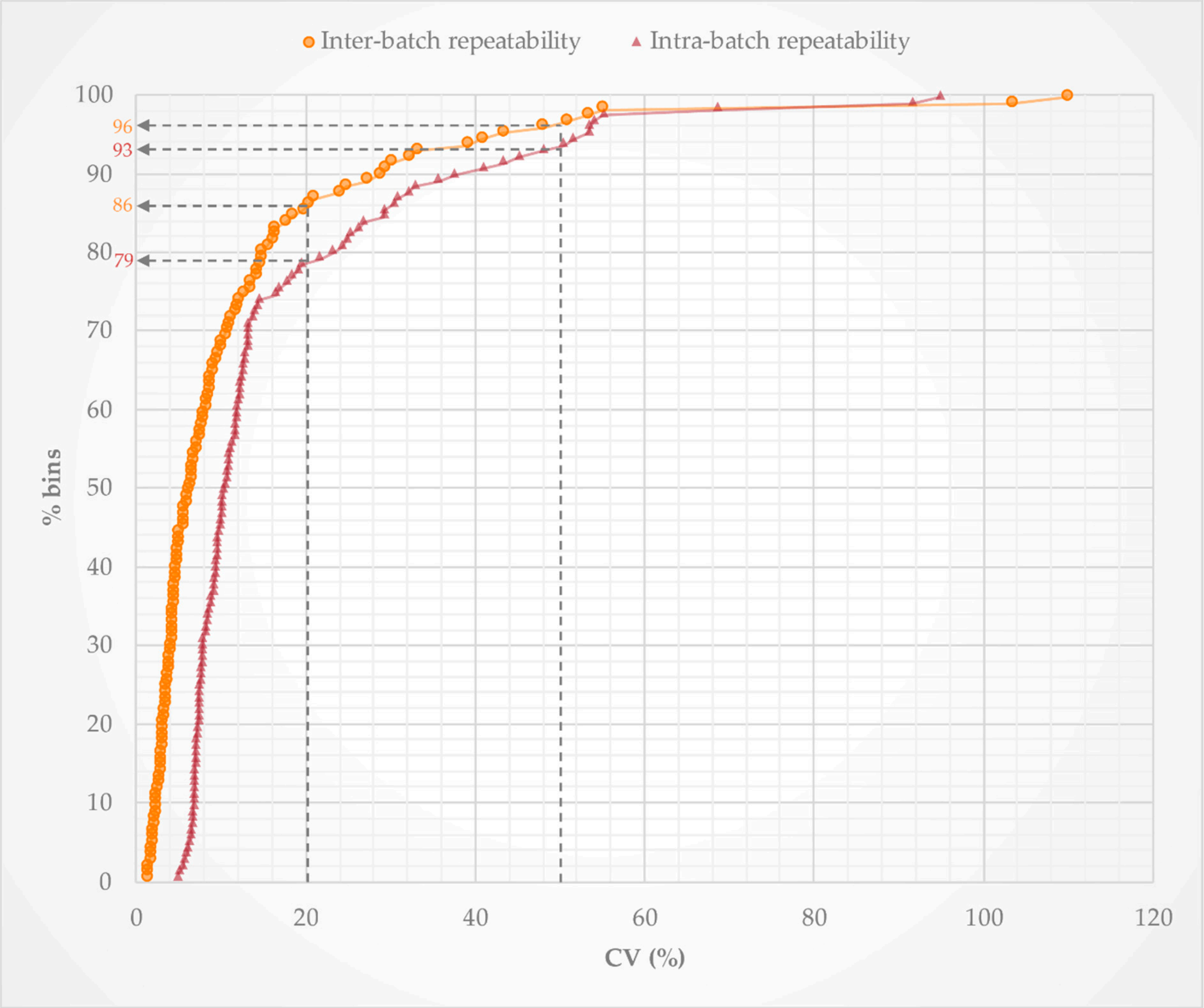

Figure S3. Inter-batch and intra-batch repeatability of all ^{1}H-NMR bins in QC samples prior to statistical analyses. FDA suggested 20% CV cut-off related to, 79% (intra-batch repeatability) and 86% (inter-batch repeatability) of all bins, while selecting a 50% CV cut-off, 93% (intra-batch repeatability) and 96% (inter-batch repeatability) of all bins fell within range.

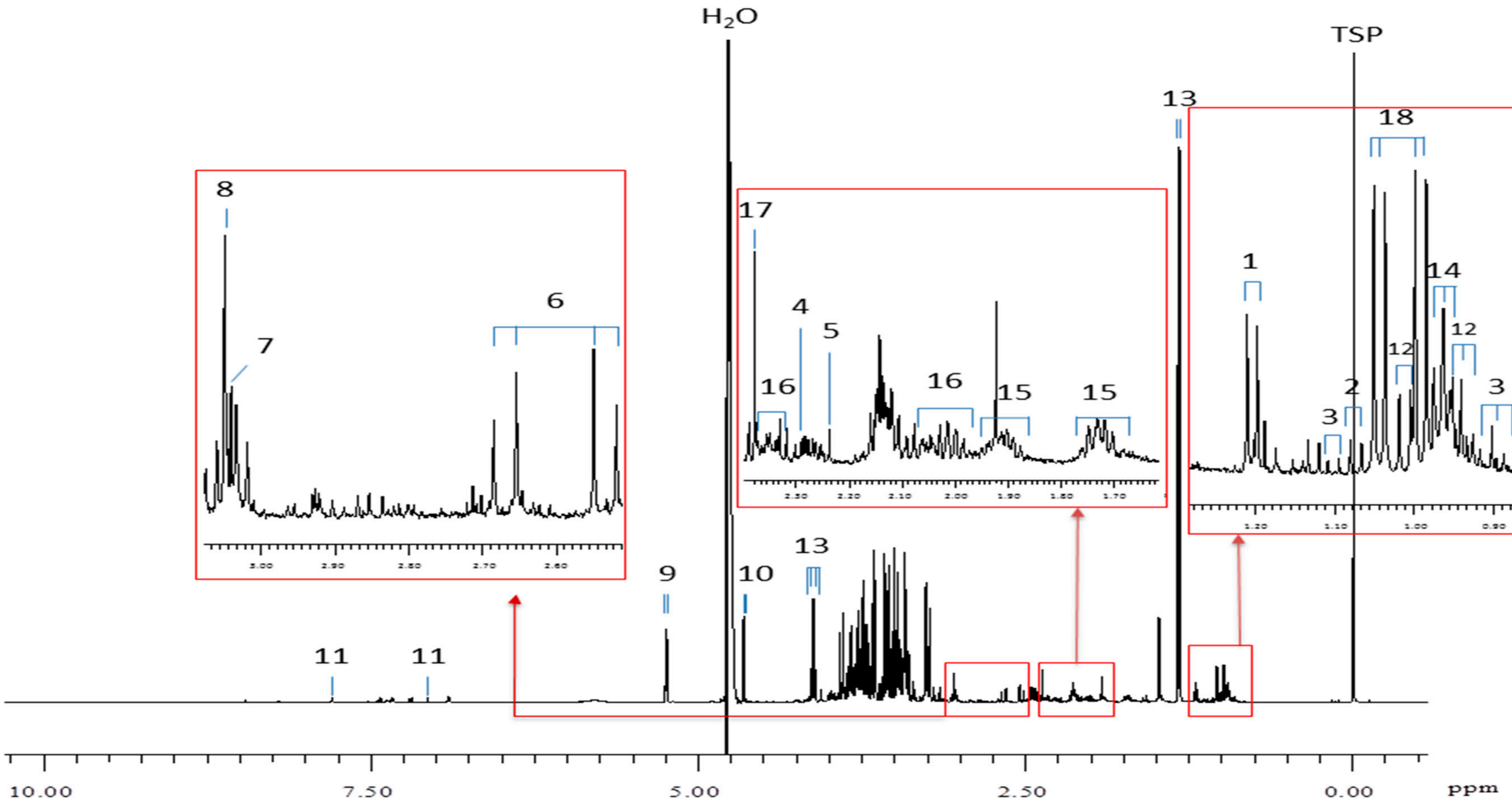

Figure S4. 1D ^{1}H-NMR spectrum of QC sample with important metabolites identified. 1=3-Hydroxybutyric acid (1.21d, J=6.2Hz), 2=3-Hydroxyisobutyric acid (1.08d, J=7.0Hz), 3=3-Methyl-2-oxovaleric acid (0.90t, J=7.4Hz; 1.10d, J=7.0Hz), 4=Acetoacetic acid (2.28s), 5=Acetone (2.24s), 6=Citric acid (2.60AB, J=15.3Hz), Creatine (3.04s; 3.93s), Creatinine (3.05s; 4.06s), 9=α-Glucose (5.24d, J=3.7Hz), 10=β-Glucose (4.66d, 7.9Hz), 11=Histamine (7.06s, 7.79d), 12=Isoleucine (0.94t, J=7.4Hz; 1.01d, J=7.0Hz), 13=Lactic acid (1.33d, J=7.0Hz; 4.12q, J=6.9Hz), 14=Leucine (0.96dd, J=5.9Hz), 15=Lysine (1.73m; 1.91m; 3.02t, J=7.6Hz), 16=Proline (2.01m), 17=Pyruvic acid (2.38s), 18=Valine (0.99d, J=7.0Hz; 1.04d, J=7.0Hz).

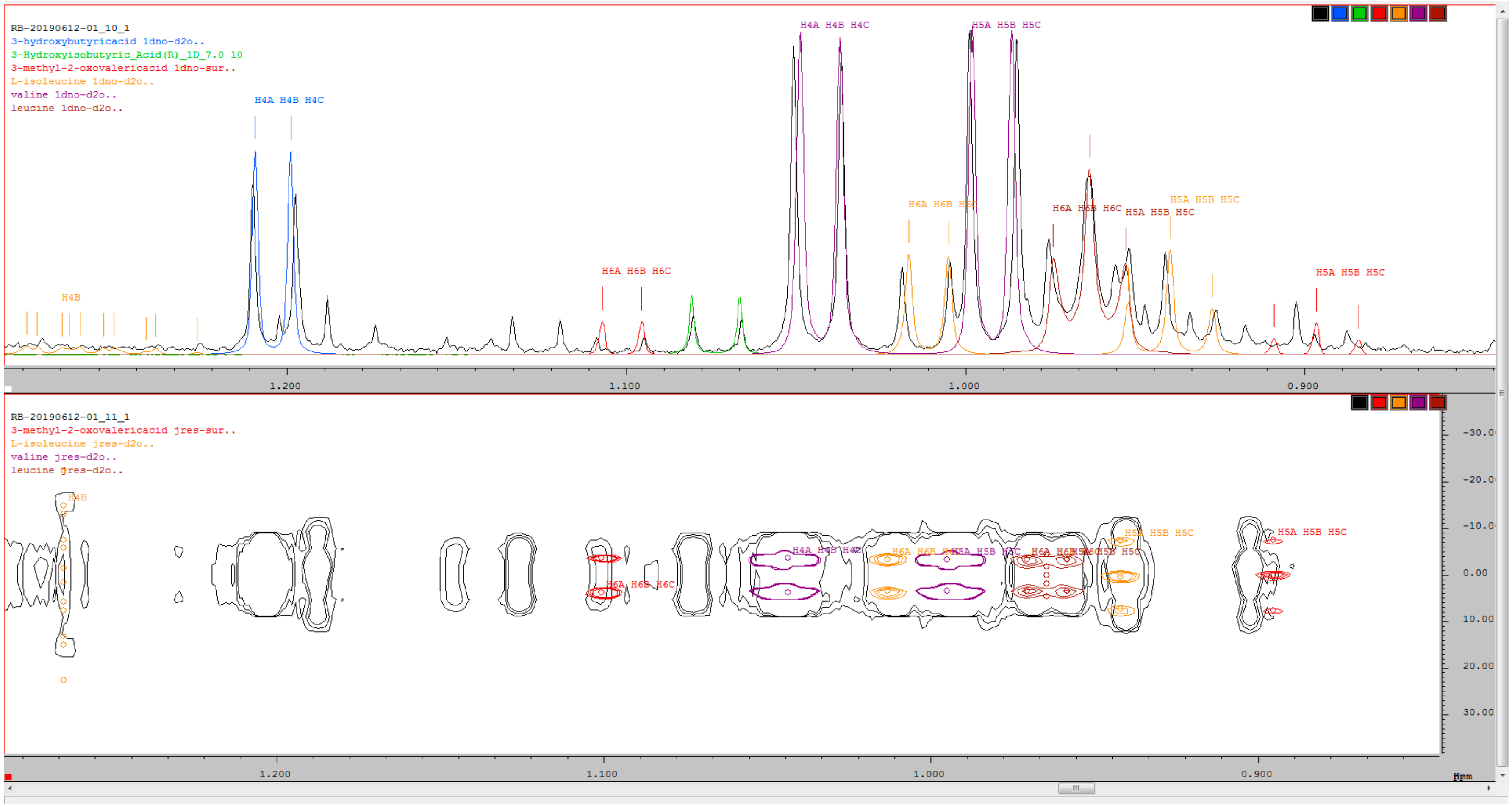

Figure S5. 1D ^{1}H-NMR and 2D ^{1}H-^{1}H JRES NMR confirmation of metabolites using pure compound library. Metabolites: 3-hydroxybutyric acid, 3-hydroxyisobutyric acid, 3-methyl-2-oxovaleric acid, isoleucine, valine, and leucine.

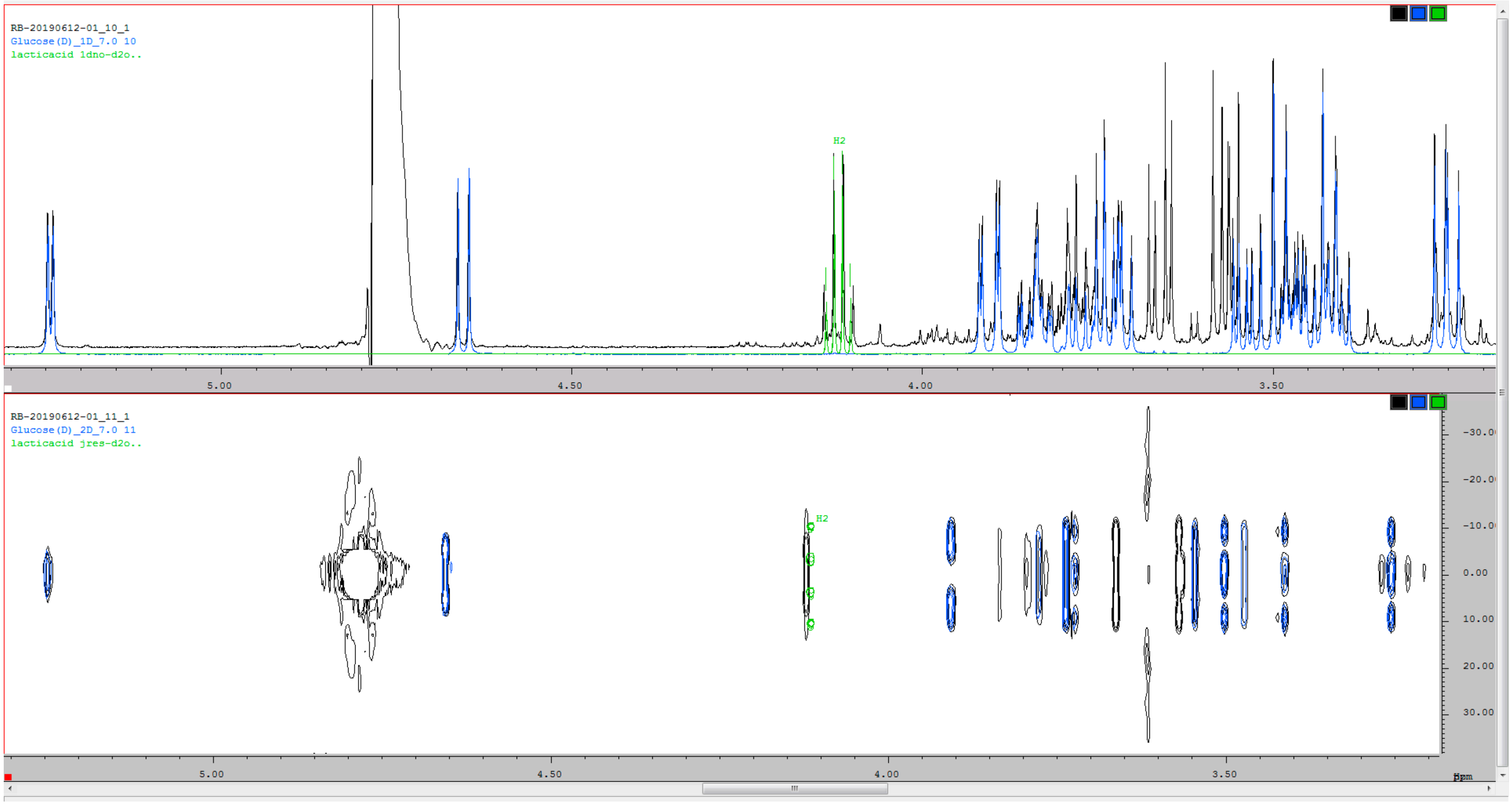

Figure S6. 1D ^{1}H-NMR and 2D ^{1}H-^{1}H JRES NMR confirmation of metabolites using pure compound library. Metabolites: glucose and lactic acid.

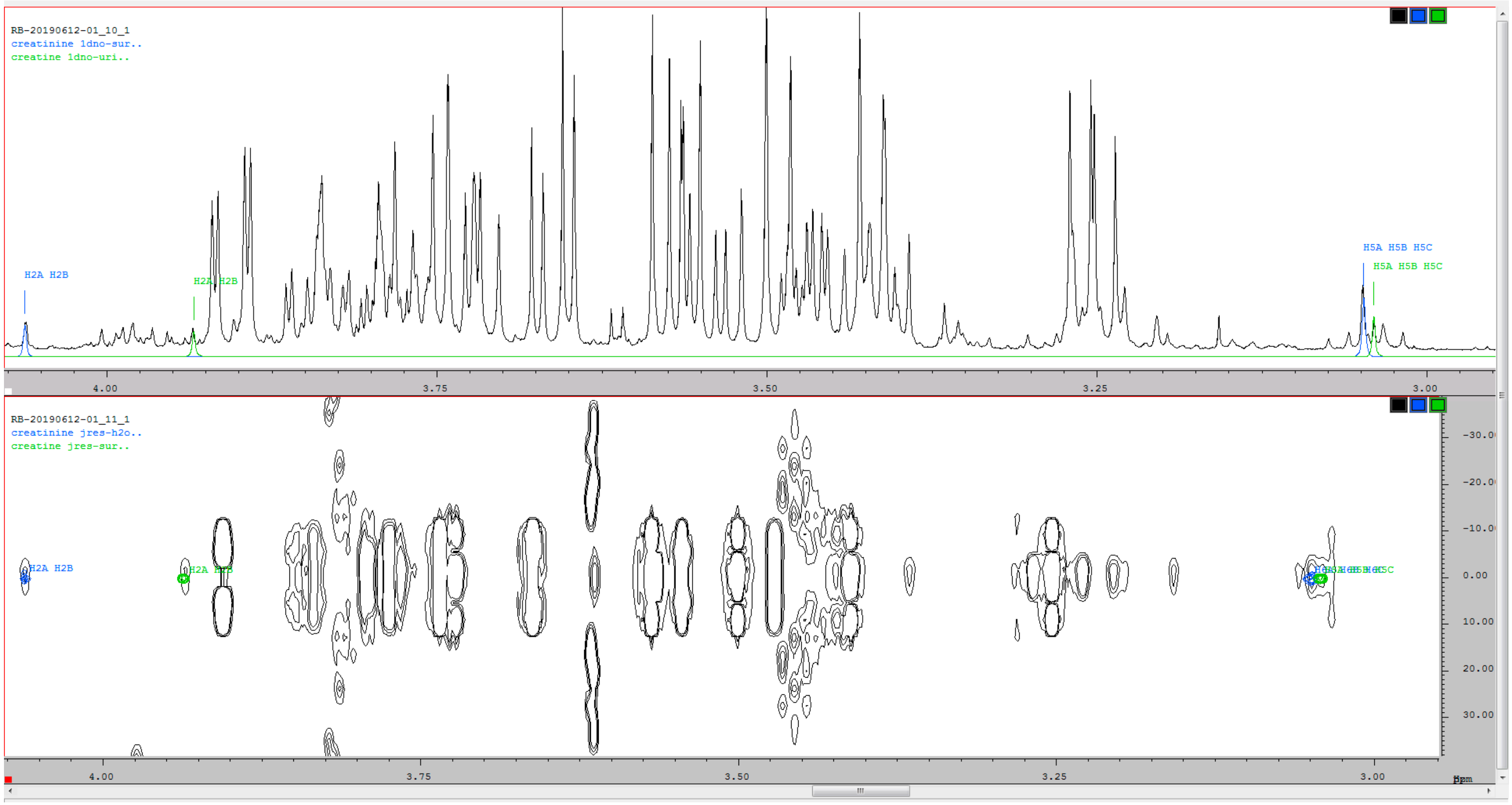

Figure S7. 1D ^{1}H-NMR and 2D ^{1}H-^{1}H JRES NMR confirmation of metabolites using pure compound library. Metabolites: creatinine and creatine.

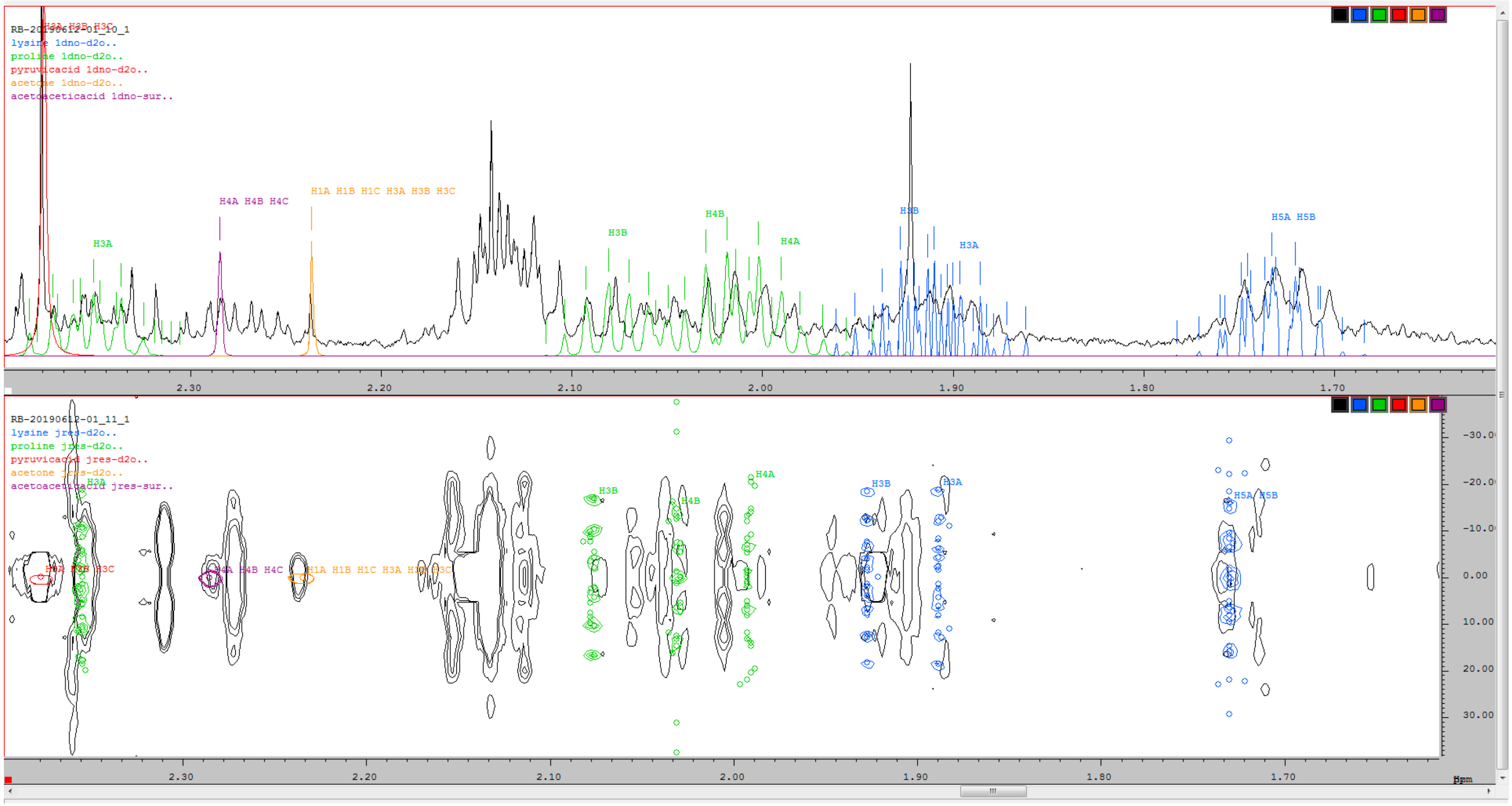

Figure S8. 1D ^{1}H-NMR and 2D ^{1}H-^{1}H JRES NMR confirmation of metabolites using pure compound library. Metabolites: lysine, proline, pyruvic acid, acetone, and acetoacetic acid.

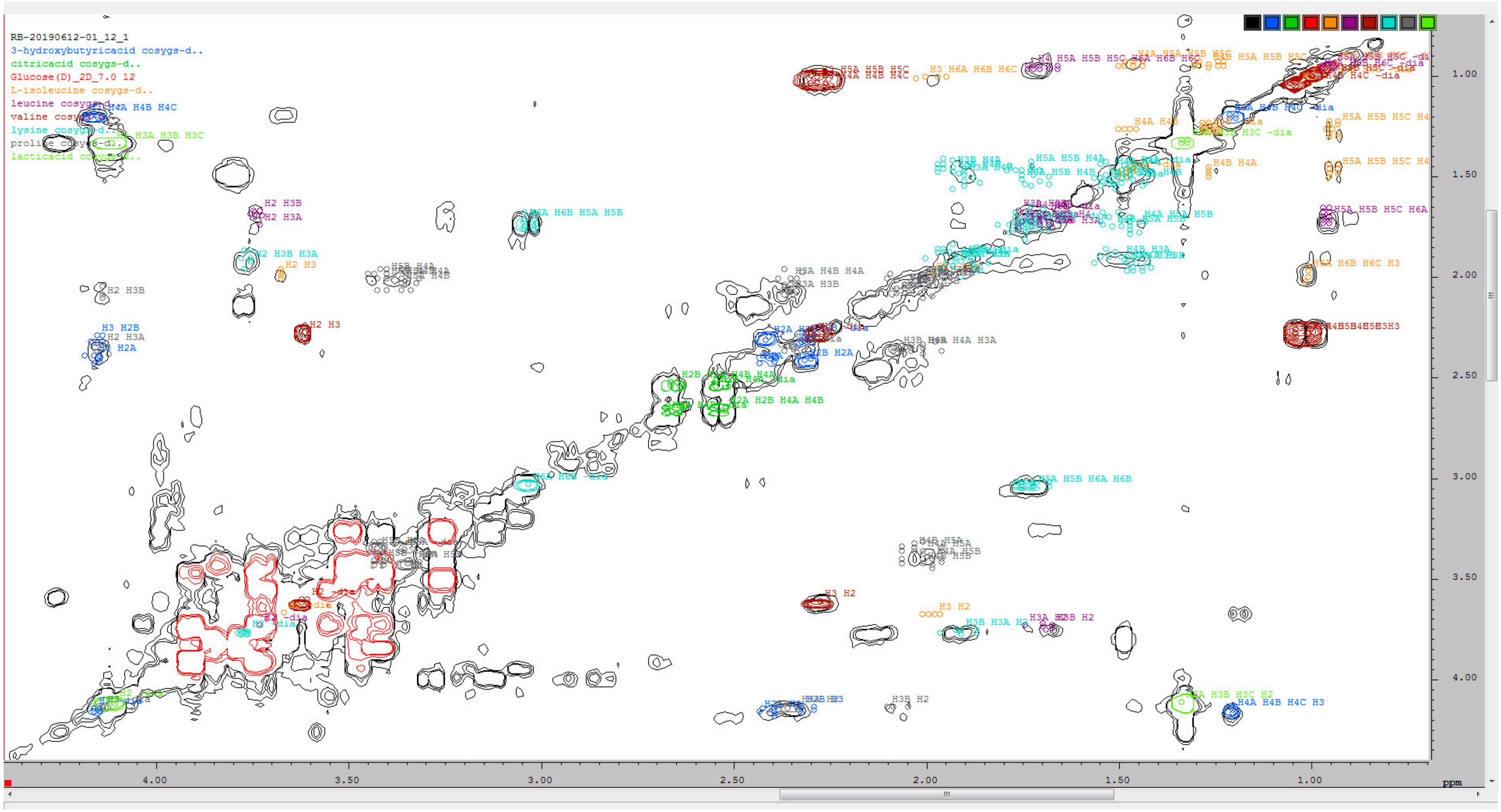

Figure S9. 2D ¹H-¹H COSY NMR confirmation of metabolites using pure compound library. Metabolites: 3-hydroxybutyric acid, citric acid, glucose, isoleucine, leucine, valine, lysine, proline, and lactic acid.
